# Physical therapy interventions for people experiencing homelessness to improve pain and self-perceived health status

**DOI:** 10.1186/s12889-024-18453-6

**Published:** 2024-04-09

**Authors:** Carolina Jiménez-Sánchez, Natalia Brandín-de la Cruz, Raquel Lafuente-Ureta, Marina Francín-Gallego, Sandra Calvo, Rocío Fortún-Rabadán, Sara Pérez-Palomares

**Affiliations:** 1 Department of Physical Therapy, Faculty of Health Sciences, Universidad San Jorge, Villanueva de Gállego, Zaragoza, Spain; 2 IIS Aragón, Zaragoza, Spain; 3 Department of Physiatry and Nursing, Faculty of Health Sciences, University of Zaragoza, Zaragoza, Spain

**Keywords:** Homelessness, People experiencing homelessness, Musculoskeletal disorders, Pain, Self-perceived health, Self-rated health, Physical therapy

## Abstract

**Background:**

Homeless shelters have emerged as components of the social services network, playing an important role in providing health care to the homeless population. The aim of this study was to evaluate an individualized physical therapy intervention for people experiencing homelessness and to determine the relationship between self-perceived variables.

**Methods:**

Pre and post study, setting at the “Santa y Real Hermandad de Nuestra Señora del Refugio y Piedad” homeless shelter in Zaragoza, Spain. Participants were people experiencing homelessness with musculoskeletal disorders who attended a physical therapy service at shelter facilities**.** A physical therapy program was implemented including health education, exercise and manual therapy, electrotherapy, thermotherapy and bandaging. Demographic variables (age and gender), nationality, employment situation, educational level, pain location, number of painful areas, feeling of loneliness (3-Item Loneliness Scale; values from 3 to 9), pain intensity (Numerical Pain Rating Scale [NPRS]; from 0 to 10) and self-perceived health (Clinical Global Impression [CGI]; from 1 to 7).

**Results:**

Sixty-four homeless people (age of 46.4 ± 10.9 years) participated in the study. Musculoskeletal pain was reported by 98.4% of subjects, with moderate pain intensities (6.1), and 48.4% presenting with pain at multiple sites. Perceptions of loneliness were low (3.7 ± 2.5) and self-perceived health status was moderately ill (3.5 ± 1.7). Positive significant correlations were identified between pain intensity and self-perceived health. The average number of sessions was 1.5 (± 0.8), with manual therapy (35.6%) followed by health education (23.5%) being the most frequently used techniques. Both pain and self-perceived health improved after treatment, even following a brief intervention.

**Conclusions:**

This study demonstrates the potentially negative impact of untreated pain on the self-perceived health of homeless individuals with musculoskeletal disorders that should be targeted for consideration. The findings suggest that a paradigm shift in pain management, including a physical therapy service in shelters, is needed to address the rehabilitation demands of these individuals in a real-life context. This study was approved by the Aragon Ethics Committee (PI19/438) and performed according to the Transparent Reporting of Evaluations with Nonrandomized Designs (TREND) statement.

## Introduction

Worldwide, 1.6 billion people live in inadequate housing conditions, and about 15 million are evicted each year [1]. The homeless population consists of people with no fixed abode, living on the streets, housed in a shelter or in some unstable or non‐permanent situation [2]. Specifically in Spain, sororities and homeless shelters have emerged as components of the social services network, playing an important role in providing health care to the homeless population. In 2022, the Spanish National Institute of Statistics estimated that there had been 28,552 cases of homeless people being treated in homeless shelters [3].

Persons experiencing homelessness (PEH) are exposed to conditions that lead to vulnerability, premature mortality, and difficulty accessing basic services [4, 5]. PEH use emergency services more frequently than other adults [6], with a significantly higher number of visits [7]. However, the low utilization of non-emergency primary health care by this population, is likely due to the differences in social status, the perception of being judged, which can lead to relational barriers, or perhaps the fact that PEH prioritizes finding shelter and food over accessing health and social services, in part because of bureaucracy and rigid hours of operation, among other factors [8].

Despite the high prevalence of musculoskeletal problems among PEH [6], specific physical therapy programs [9] are very scarce. Only two mixed-method studies have conducted a physical therapy intervention with homeless people, with a larger qualitative design focused on qualitative analysis [10, 11]. Location of services and transportation difficulties, as well as cost and waiting times, were the main barriers to accessing physical therapy services [10, 11]. It seems that providing physical therapy services directly at the shelters increase the percentage of people who completed more than one session [10], with the recommendations to work with essentials and take a more flexible approach to provide walk-in physical therapy services [11]. Only one study assessed the effectiveness of treatment based on improvement in quality of life [11].

On the other hand, few studies have focused on the study of pain in these individuals, as the presence of potentially serious pathologies and difficult access to medical services have relegated the examination of pain to the background [12]. However, it has been reported that PEH often suffer to a high degree from pain that limits them physically. PEH are known to report a greater number of barriers which are associated with increased pain intensity and subjective feelings of loneliness and helplessness, which alter subjective perceptions of their health status [13]. These include stress related to shelter life and poor sleeping conditions.

Self-perceived health (SPH) or self-rated health is a subjective measure of a person´s health status, that includes psychosocial and physical aspects as well as cultural and socioeconomic factors in the general population [13]. It is closely related to morbidity and disability, although it can also be a robust predictor of mortality and measure a global health status [13]. Among objective determinants of health, the presence of a chronic disease is one of the greatest predictors of worse self-perceived health for both females and males aged 50–64 years old [14]. Furthermore, the impact of health conditions is mediated through symptoms: the health conditions themselves are likely less important than their symptoms for determining SPH. In particular, pain is a relevant factor in those with the worst SPH [15]. Among social determinants of SPH, age plays a relevant role, but low educational level, unemployment, and low levels of physical activity are associated with poor SPH regardless of generation and gender [16–18]. This underlies the impact of social inequalities in SPH [19]. Factors associated with better SPH in PEH are being male; living abstinently; holding a health card [20]; adequate sleep; and having fewer chronic health conditions [21].

There is a gap in the literature, where authors such as Kiernan et al., propose that measurement methods should be evaluated specifically for these individuals, as well as research into appropriate rehabilitation and support interventions for this vulnerable population [22]. To the authors' knowledge, there are few studies that have performed a quantitative analysis of a comprehensive physical therapy intervention in shelters for PEH with musculoskeletal disorders; and none evaluated the SPH of these PEH in a musculoskeletal clinical context [4]. Our proposal was to develop a standard physical therapy intervention that was accessible to PEH by applying it in response to immediate demand in shelters.

The aim of this study is to evaluate an individualized physical therapy intervention for people experiencing homelessness and to determine the relationship between self-perceived variables (loneliness and helplessness, pain, and self-perceived health status).

## Material and methods

### Study design

A pre-post intervention study was performed according to the Transparent Reporting of Evaluations with Nonrandomized Designs (TREND) statement [23]. This study was approved by the Aragon Ethics Committee (PI19/438).

### Participants

Subjects eligible for this study were homeless and temporarily sleeping in a homeless shelter (“Santa y Real Hermandad de Nuestra Señora del Refugio y Piedad”) in Zaragoza, Spain. They were invited by the medical staff to participate in the study if they met the eligibility criteria. All participants provided signed informed consent before the start of the evaluation process.

Inclusion criteria were the following: 1) age over 18 years; 2) people experiencing homelessness 3) having an identifiable musculoskeletal disorder that can be treated by physical therapy according to medical criteria (for example, pain, muscle tenderness or atrophy, joint sprain, or loss of range of motion). The exclusion criteria were: 1) inability to follow instructions or cooperate in the performance of the examination, either due to language barrier or because of cognitive impairment; 2) presence of signs indicative of a medical emergency (for example, signs of fracture, acute disc extrusion, acute medullary or nerve compression); 3) presence of severe psychiatric disorders (for example, schizophrenia, bipolar disorder, substance abuse, etc.) The above criteria were assessed by medical examination at the shelter consultation and by clinical history of the homeless people.

### Variables and data measurements

Data were collected by physical therapy staff between March 2018 and February 2020 through interviews in the following standardized order.

#### Sociodemographic and clinical assessment

At the beginning of physical therapy session, sociodemographic data such as age, gender, nationality, education level, and employment situation were collected.

A clean body chart (anterior and posterior views) was used to document pain and symptom localization [24]. Subsequently, the body charts were divided into different sections to obtain two variables: (i) pain/symptom localization, a qualitative record of the name of the area(s) in which participants reported the presence of pain/symptoms (e.g., cervical spine or upper extremity), and (ii) the number of painful areas, a numerical measure of the number of different areas in which participants marked the presence of pain [24]. The multisite pain category was used when participants reported pain in more than one area [25, 26].

#### Self-perceived outcomes

Feelings of loneliness and helplessness was assessed with the Three Item Loneliness Scale. This scale has satisfactory reliability and both concurrent and discriminant validity [27]. It also explores objective and subjective social isolation and is appropriate in this context because it measures general loneliness well using three items and a simplified set of response categories [28]. The scale asks how often respondents feel they have no company; feel excluded; and feel isolated from others. Items are rated on a scale of 1 (almost never) to 3 (often) and summed to a score of 3 to 9, with higher scores reflecting greater loneliness [29].

The intensity of musculoskeletal pain was assessed with the 11-point Numerical Pain Rating Scale (NPRS) [24], commonly used to assess pain with scores from 0 to 10 and definitions of 0 = no pain and 10 = worst pain imaginable. Participants were asked to rate the mean pain intensity to evaluate the current pain experience. Self-perceived health status was assessed using the Clinical Global Impression Scale (CGI). The CGI is a classic instrument used to assess psychiatric disorders with strong validity, although it has also been used in other clinical settings [30]. The CGI asked participants to assess their self-perceived health status at that time. The CGI comprises two companion one-item measures with a 7-point Likert rating scale evaluating the following: (a) independent severity of illness (assessment of a patient's current symptom severity) (CGI-S) from 1 to 7, where 1 = normal, not ill, 2 = borderline ill, 3 = minimally ill, 4 = moderately ill, 5 = markedly ill, 6 = severely ill, and 7 = among the most extremely ill patients; and (b) change from the initiation of treatment (CGI-I) on a similar seven-point scale, where 1 = very much improved, 2 = much improved, 3 = minimally improved, 4 = no change, 5 = minimally worse, 6 = much worse and, 7 = very much worse [31, 32]. The question used for the CGI-S was: “*How sick do you currently feel?* and for the CGI-I: “*How much change have you felt after the received care?”.* The CGI will be classified into the following categories based on CGI-S scores: normal (1), low (2–3), moderate (4), and severe (5–7) self-perceived health status.

As these are subjective assessments, the assessors were trained to homogenize the explanations they give to the patients.

#### Treatment satisfaction

To assess satisfaction with the physical therapy program, participants were presented with an *ad-hoc* 5-point Likert questionnaire, between 1 = none and 5 = very much.

The questions were: a) General: 1) The intervention performed was appropriate for me; 2) The professionalism of the physical therapists was appropriate; and 3) The attitude, interest, and treatment of the physical therapists were appropriate. Physical factors were assessed in section b): 4) I experienced an improvement in my general condition; 5) My pain, tension, etc. reduced. Psychological factors were assessed in section c): 6) I gained personal satisfaction from participating in this project; 7) My expectations from the intervention carried out were met; and d) Educational factors: 8) I increased my knowledge about health; and 9) I will be able to use the acquired knowledge later.

### Procedure

Patients were treated in the shelter facilities. The physical therapy program took place twice a week during the medical consultation hours. The number of sessions was based on the length of stay in the facility, which was a maximum of 1 month for each person (maximum of eight sessions). The sessions lasted about 20 to 30 min, depending on the needs identified and the type of therapy carried out.

To determine the appropriate type of intervention, a routine examination was performed in which functional tests were performed, such as active movement without pain, final sensation during passive movement, translational joint play, muscle assessment (length, and strength per the Medical Research Council Scale), and provocation-relief tests.

The number and type of techniques used were recorded.

### Intervention

The intervention was carried out by 5 physical therapists trained in musculoskeletal physical therapy and based on standard physical therapy sessions.

According to the results of the assessment, different physical therapy techniques were selected and applied for each subject, who could receive one or more interventions in the same session: 1) Health education, with analysis of postural habits and promoting awareness behaviors [33]; 2) Exercise therapy, featuring movement as a therapeutic intervention and focusing on therapeutic exercise to recover flexibility, strength and endurance [34]; 3) Manual therapy, for the treatment of musculoskeletal pain [35]; 4) Electrotherapy using transcutaneous electrical nerve stimulation (TENS) as an analgesic technique [36]; 5) Thermotherapy, or the application of heat with Infra-Red (IR) light to increase blood circulation in the affected areas [37], and 6) Bandaging with functional and neuromuscular bandages as required [38].

### Statistical analysis

Statistical analysis was conducted using IBM SPSS Statistics for Windows, Version 28.0 (IBM Corp, Armonk, NY, USA), and SigmaPlot, Version 14.0 (Systat Software, Canada). For the descriptive analysis, numbers (percentages), mean and standard deviation (SD), or median and interquartile range were used.

The normality of the quantitative data was checked for using the Shapiro–Wilk test.

Differences between variables by nationality and gender were analyzed using the Mann–Whitney U test or the Chi-square test, depending on the nature of the variables.

Spearman’s rank correlations were carried out to examine the relationship between variables. The strength of correlations was interpreted as either low (0.00 – 0.25), fair (0.26 – 0.50), moderate to good (0.51 – 0.75), and good to excellent (> 0.75) [39].

The Wilcoxon test was applied to highlight the differences between pre- and post-intervention data measurements. The Cohen effect size was also calculated with the following interpretation: trivial (< 0.2), small (0.2 to 0.5), moderate (> 0.5 to 0.8), and large (> 0.8) [40].

The statistical analysis was conducted at a 95% confidence level. Statistical significance was set at *p* < 0.05.

## Results

**Fig. 1:**
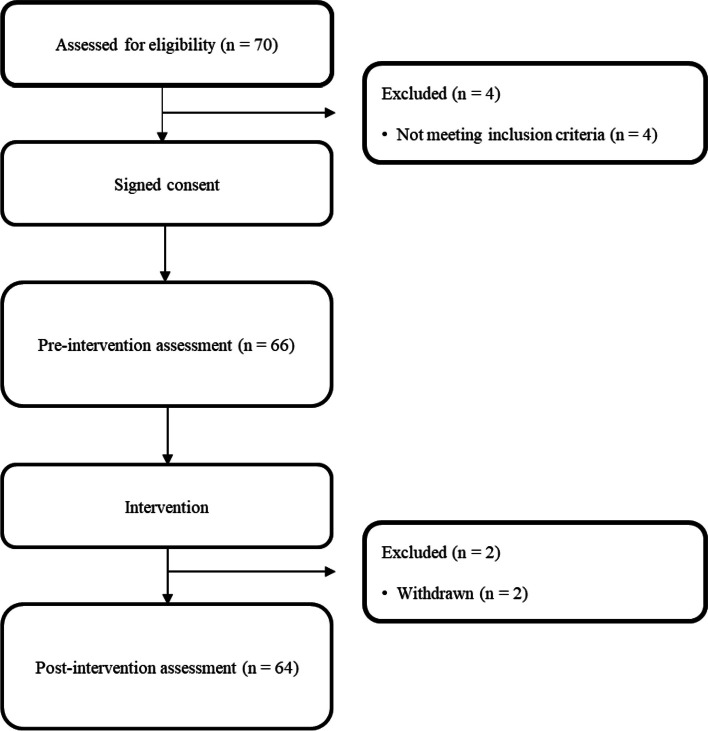
Flowchart of the study

Sixty-four homeless individuals who required physical therapy were included in this study (Fig. 1). Almost all participants had musculoskeletal pain without any other serious diagnosis, except for three individuals with pain due to stroke, knee sprain, and ankle fracture, respectively.

### Baseline sociodemographic and clinical characteristics

Baseline and clinical demographic characteristics of the participants by gender and nationality are shown in Table 1. No statistically significant differences were found between genders and nationalities (*p* > 0.05).

**Table 1:** Demographic and baseline clinical characteristics of the study participants

		**Total** ***n* = 64**		**Spanish** ***n* = 35**		**Non-Spanish** ***n* = 29**	
		**Male**	**Female**	**Male**	**Female**	**Male**	**Female**
**Sex n (%)**		60 (93.75%)	4 (6.25%)	33 (94.29%)	2 (5.71%)	27 (93.10%)	2 (6.90%)
**Age (years)**		46.5 ± 11.3 47 [38.5–55.5]	44.8 ± 3.8 45 [42–47.5]	48. 9 ± 11.9 52 [39.8–57.8]	44.5 ± 6.4 44.5 [40–49]	43.6 ± 9.8 44 [37.3–47]	45 ± 1.4 45 [44–46]
		46.4 ± 10.9 46.5 [39.5–54.5]		48.7 ± 11.6 51 [40–57]		43.7 ± 9.5 44 [37.8–47]	
**Employment status** **n (%)**	Unemployed Temporarily-employed Retired Disability	39 (60.94%) 11 (17.19%) 4 (6.25%) 4 (6.25%)	2 (3.13%) 2 (3.13%) 0 (0%) 2 (3.13%)	18 (51.43%) 8 (22.86%) 3 (8.57) 4 (11.43%)	2 (5.71%) 0 (0%) 0 (0%) 0 (0%)	21 (72.41%) 3 (10.34%) 1 (3.45%) 0 (0%)	0 (0%) 2 (6.90%) 0 (0%) 2 (6.90%)
**Numerical Pain Rating Scale (NPRS)**		6.0 ± 2.1 6.0 [5–8]	5.8 ± 2.8 6.5 [2–8]	6.0 ± 2.1 6.0 [4–8]	6.5 ± 2.1 6.5 [5–8]	6.1 ± 2.0 6.0 [5–8]	5.0 ± 4.2 5.0 [2–8]
		6.1 ± 2.1 6.0 [5–8]		6.0 ± 2.1 6.0 [4.3–8]		5.9 ± 2.1 6.0 [5–8]	
**Symptom location** **n (%)**	Cervical spine Thoracic spine Lumbar spine Upper limbs Lower limbs	14 (23.3%) 11 (18.3) 11 (18.3) 14 (23.3%) 22 (36.7%)	1 (25%) 1 (25%) 1 (25%) 1 (25%) 1 (25%)	9 (27.3%) 7 (21.2%) 5 (15.2%) 7 (21.2%) 13 (39.4%)	0 (0%) 0 (0%) 0 (0%) 1 (50%) 1 (50%)	5 (18.5%) 4 (14.8%) 6 (22.2%) 7 (25.9%) 9 (33.3%)	1 (50%) 1 (50%) 1 (50%) 0 (0%) 0 (0%)
**Multisite pain n (%)**		28 (46.7%)	3 (75%)	14 (42.4%)	2 (100%)	14 (51.9%)	1 (50%)
		31 (48.4%)		16 (45.7%)		15 (51.7%)	
**Number of painful areas**		1.6 ± 0.8	1.8 ± 0.5	1.6 ± 0.9	2.0 ± 0	1.6 ± 0.6	1.5 ± 0.7
		1.6 ± 0.9		1.6 ± 0.9		1.6 ± 0.6	
**Three-Item Loneliness Scale**		3.6 ± 2.5 3.0 [2–4]	4.5 ± 3.0 3.0 [3–6]	3.9 ± 2.7 3.0 [2–6]	3.0 ± 0 3.0 [3–3]	3.3 ± 2.2 3.0 [1–4]	6.0 ± 4.2 6.0 [3–9]
		3.7 ± 2.5 3.0 [2–4]		3.8 ± 2.7 3.0 [2–5.5]		3.4 ± 2.3 3.0 [1–4]	
**Clinical Global Impression Scale (CGI-S)**		3.5 ± 1.6 4.0 [2–5]	4.3 ± 2.4 5.0 [2.5–6]	3.5 ± 1.7 4.0 [2–5]	3.5 ± 3.5 3.5 [1–6]	3.4 ± 1.6 4.0 [2.2–4.8]	5.0 ± 1.4 5.0 [4–6]
		3.5 ± 1.7 4.0 [2–5]		3.5 ± 1.7 4.0 [2–5]		3.6 ± 1.6 4.0 [2.7–5]	

Data are presented as number (percentage); mean ± standard deviation; and median [interquartile range]

The mean age of participants was 46.4 ± 10.9 years (range: 39.5 to 54.5 years), with four (6.2%) females (age 44.8 ± 3.8 years) and 60 (93.8%) males (age 46.5 ± 11.3 years). Regarding nationality, most (54.7%) had been born in Spain, followed by Algeria (12.5%), Colombia (9.4%), Morocco (6.3%), Romania (6.3%), or other countries (10.8%). All participants had no qualifications or had only completed primary education. Of all participants, 41 (64.04%) were unemployed, 13 (20.3%) had temporary employment, four (6.3%) were retired, and six (9.4%) had a disability (Table 1).

Sixty-three participants (98.4%) reported musculoskeletal pain at baseline with a mean intensity of 6.1 ± 2.1, and 44% of participants reported severe pain (**≥ **7/10 in the NPRS).

The body region most affected by musculoskeletal pain was the lower limbs (35.9% of participants), followed by the cervical spine (23.4%), upper extremities (23.4%), and finally lower back (18.8%) and chest (18.8%). 48.4% reported multisite pain. The mean number of painful areas for each participant was 1.6 ± 0.8 (Table 1).

Regarding health status at baseline, 54.6% of participants scored 3 or higher on the CGI-S scale, with a mean score value of 3.5 ± 1.7 (moderately ill), indicating poor self-perceived health status in the sample. Although females reported worse health status, with a status of "severely ill" as opposed to a status of "moderately ill" in men, this difference was not statistically significant (*p* > 0.05). Self-perceived health status did not differ between Spanish and non-Spanish individuals.

Regarding perceptions of loneliness and helplessness, feelings of loneliness were not prevalent in this study sample. The mean score on the three-item loneliness scale was 3.7 ± 2.5, with females scoring higher. Spanish participants had a higher score (+ 0.4) than participants born elsewhere (Table 1).

## Correlations

A moderate to good statistically significant correlation was observed between pain intensity (NPRS) and perception of health status (CGI-S). More severe musculoskeletal pain was associated with a worse perception of health status at baseline (rho = 0.539, *p* < 0.001).

There were no other significant correlations among the other variables (*p* > 0.05).

When the ranges of health state perception scores were compared with pain intensity, the highest pain intensity scores were found for moderate and severe CGI-S scores (Table 2).

**Table 2:** Comparison of pain intensity and self-perceived health

n	CGI-S	NPRS
12 (18.8%)	Normal (1)	5.5 ± 1.9 5.5 [4–7]
17 (26.6%)	Low (2–3)	4.3 ± 2.4 4 [3–5.25]
15 (23.4%)	Moderate (4)	6.3 ± 1.5 6 [6, 7]
20 (31.2%)	Severe (5–7)	7.5 ± 1.6 8 [7, 8]

*Abbreviations*: *CGI-S* Clinical Global Impression-Severity, *NPRS* Numerical Pain Rating Scale

Data are presented as a number (percentage); mean ± standard deviation; and median [interquartile range]

### Changes to NPRS and CGI following physical therapy intervention

A total of 230 physical therapy interventions were performed, with manual therapy being the most used (82 interventions), followed by health education (54 interventions), bandages (36 interventions), thermotherapy (32 interventions), exercise therapy (17 interventions), and electrotherapy (9 interventions).

Overall, the average number of interventions for each participant was 1.5 ± 0.8 sessions: 59.4% of participants received one session (*n* = 38); 34.4% (*n* = 22) received two sessions; 3.1% (*n* = 2) received three sessions; and 1.6% (*n* = 1) received five sessions. The number of sessions each participant received was unrelated to baseline variables or pre-and post- intervention variables. There was also no difference between participants who attended only one intervention (59.4%) and those who attended more than one (40.6%).

Despite not being statistically significant, all females (*n* = 4) attended only one session. The attendance of Spanish and non-Spanish subjects was similar (*p* > 0.05).

**Fig. 2:**
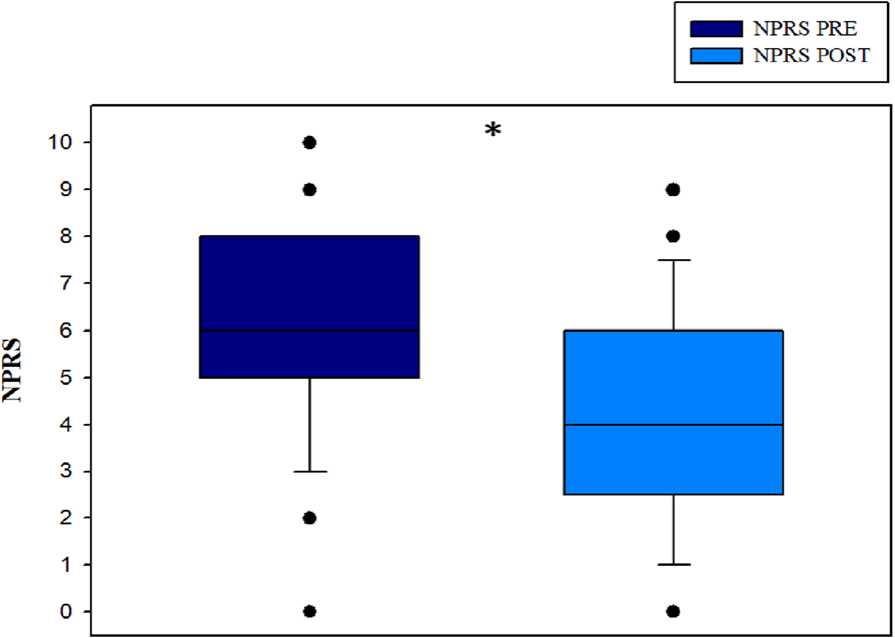
Comparison of pain intensity (0–10 NPRS). Abbreviations: NPRS: Numerical Pain Rating Scale; PRE: Pre-intervention; POST:Post‑intervention. Using the Wilcoxon test. *p < 0.001

Regarding pain intensity according to the NPRS, the mean score at the end of treatment was 4.2 ± 2.3 (median 4, IQR 2.8–6). There was a statistically significant reduction of 1.9 ± 1.7 points (*p* < 0.001) with a moderate magnitude in terms of effect size (d = 0.633) (Fig. 2).

**Fig. 3:**
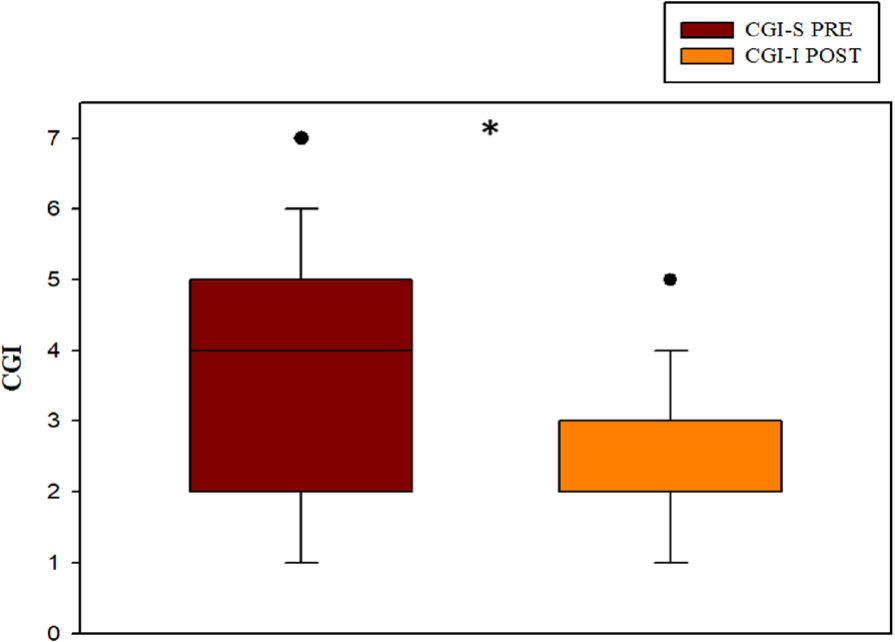
Comparison of self-perception of health status (1–7 CGI). Abbreviations CGI-S: Clinical Global Impression-Severity CGI-I: Clinical Global Impression-Improvement PRE: Pre-intervention POST: Post‑intervention. Using the Wilcoxon test. * *p* < 0.05

When considering the whole sample, there was a statistically significant improvement in mean self-perceived health status of 0.9 ± 2.0 points (*p* = 0.005) from moderately ill (3.5 ± 1.7; median 4, IQR 2–5) to moderately better (2.5 ± 1.1; median 2, IQR 2–3). The effect size indicated a small difference between the baseline score and the score after the physical therapy intervention (d = 0.352) (Fig. 3). A strong correlation was found, indicating greater improvement in self-perceived health among those who reported poorer health status at baseline (rho = 0.84, *p* < 0.001), and a moderate association with relief of musculoskeletal pain (rho = 0.41, *p* < 0.01).

Although not statistically significant, females reported greater positive effects of the intervention compared with males, both in terms of self-perceived health status and musculoskeletal pain intensity (*p* > 0.05). However, Spanish, and non-Spanish participants experienced similar improvements after the intervention with no differences (*p* > 0.05).

### Treatment satisfaction

The mean evaluation score of the intervention and the professionalism of the physical therapy team was 4.7 ± 0.3 out of 5. In relation to physical, psychological, and educational factors, the mean scores were 4.6 ± 0.4, 4.7 ± 0.3, and 4.5 ± 0.4, respectively, out of 5.

## Discussion

This is the first study to specifically examine self-perceived variables in PEH attending a physical therapy consultation at shelter facilities which also evaluated the effectiveness of physical therapy interventions. Sixty participants (93.8%) were male and four (6.2%) were female, with an overall mean age of 46.4 years. Pain at baseline was present in 98.4% of participants. The level of basal pain intensity was moderate (6.1). Pain at multiple sites was reported by 48.4% of participants. At baseline, there was a low perception of loneliness (3.7) and a moderate perception of illness (3.5). Homeless individuals who were not born in Spain did not have greater pain, were not lonelier, and did not have worse self-perceived health than Spanish PEH. There was a significant association between pain and self-perceived health status.

### Pain

Of the selected participants, all but one reported musculoskeletal pain, and in almost half of them the pain was severe, with pain intensity equal or greater than 7 points on the NRPS. We found lower intensity of pain compared to the other studies of PEH, which were focused on chronic pain [12, 41]. The most affected body regions were the lower limbs, followed by the spine, which is consistent with previous findings and may be due to the lack of a permanent residence, which means having to stand and walk for long periods and carry the weight of one´s belongings [12, 42]. Both the intensity and location of pain are important variables in the study of people living with pain. In particular, the presence of pain at multiple locations is considered to be a prognostic factor for chronic conditions [43, 44]. The present study provides new data on this: multisite pain was present in 48.4% of patients, which is higher than the figures obtained in the general population and similar to the elderly [45]. Pain improvement after therapy was at the limit of minimal clinically relevant change (1.9 ± 1.7 points), but significant, even after a short treatment (many cases only received one session). The same outcome measurement was used by Oosman [11], the only study that has reported differences in pain before and after a session of physical therapy, where pain intensity was assessed as a subdomain of the EQ-5D-5L and minimal clinically non-relevant changes were noted after treatment (0.25 points in the pain domain of 5D-QoL). The results of our study highlight the need to identify and treat pain in this underserved population from another approach. Pharmacological treatments in this group may not be a *princeps treatment* due to difficulties in prescribing psychotropic drugs in PEH, who are especially vulnerable to the mishandling of this type of substance. Pain cannot be disregarded in this population, as they can equally benefit from therapeutic strategies, especially given the extreme impact of pain and poor health in this vulnerable group.

### Self-perceived health

Poor health perception was reported by most participants irrespective of age, nationality, or gender; worse health correlated with higher pain intensities. We observed poorer perceived health status in our sample compared with the limited research that exists measuring self-perceived health among homeless people. We reported 54.6% with poor or very poor SPH (moderate and severe illness perception) compared to the 39.7% with poor or very poor SPH in a national survey study of 2,437 adults in Spain [20], or the 76% with good or very good SPH of 244 homeless adults living in an emergency shelter in Dallas [46]. Neither of these studies were performed in a clinical context, and our question differed because it related to the perception of severity of poor health when being in a health consultation and was not asked in a positive way (the question was made in terms of illness and not in terms of health). In another study of 421 individuals in Los Angeles (USA), it was found that self-rated general health status of PEH improved when they moved to permanent supportive housing. However, this was not the case if they suffered from physical limitations that did not improve until six months after moving to permanent supportive house, and poor self-perceived health was related to the number of chronic health conditions [21]. Of note in our study, not only was higher pain intensity associated with poorer SPH, but its improvement was also associated with lower perceived pain intensity after treatment.

### The physical therapy intervention

A total of 230 physical therapy interventions were carried out. Manual therapy (35.6%) and health education (23.5%) were the most frequently used, the median number of sessions was 1.5 (± 0.8). It can be difficult to assess the impact of physical therapy treatments in PEH due to their unstable lifestyle, which makes it difficult to schedule sessions at a specific location, resulting in irregular attendance. This is an important aspect of the PEH condition that may affects the usual form of health care. However, the only two studies which evidence physical therapy interventions used the form of scheduled sessions, although the details of the interventions are not clearly explained. In our study 41% received at least two sessions despite not providing fixed appointments and left it to the demand or need of the patients themselves and/or the circumstances of their stay in the accommodation. Our design follows the recommendations of Oosman [11], who uses a non-schedule-based care model. It seems that the accessibility of place of care facilities is more important than the number of scheduled sessions in increasing participation. It is likely that rehabilitative needs are not being met through traditional models of care [47].

Our main intervention was manual therapy (35.65%) followed by health education, in contrast to Dawes [10] and Oosman [11], where health education and self-care strategies (advice and exercise), respectively, were most frequently used. The type of technique or treatment does not appear to have affected adherence, as the number of sessions was similar to those reported by Oosman [11] or Dawes [10] (both with a median of 2 visits per patient), with the location of use being more important than the type of treatment applied. As mentioned above, the type of intervention may have influenced the relevant improvement in pain observed in our study.

### Limitations

This study has several limitations that we would like to highlight. Given the subjective nature of the assessment tools used, a qualitative assessment would have been interesting to contrast and complement the quantitative results obtained. We did not collect information on comorbidities or psychosocial factors that may affect pain severity and self-perceived health. We did not specifically evaluate neuropathic pain. The post-treatment evaluations have been adapted to the reality and availability of the PEH, so they had to be carried out just after the treatment session. Due to the multiplicity of consultations and taking into account the already expressed difficulty for this population to attend another new consultation, we have neither been able to solve the limitation of not using of separate researcher to administer the questionnaires. Further studies are needed to be conducted in different shelters in Spain, with a larger sample size, with a control group and a follow-up.

## Conclusions

This study showed a significant improvement in pain and self-perceived health status after a physical therapy intervention, as well as a relationship between these variables. These results highlight the importance of including physical therapy in an accessible model of care service. Further research is needed to evaluate a specific approach to pain management from various perspectives that should be considered in this population.

## Data Availability

The datasets used and/or analysed during the current study are available from the corresponding author on reasonable request.
